# Space Use Variation in Co-Occurring Sister Species: Response to Environmental Variation or Competition?

**DOI:** 10.1371/journal.pone.0117750

**Published:** 2015-02-18

**Authors:** Claire M. S. Dufour, Christine Meynard, Johan Watson, Camille Rioux, Simon Benhamou, Julie Perez, Jurie J. du Plessis, Nico Avenant, Neville Pillay, Guila Ganem

**Affiliations:** 1 Institut des Sciences de l’Evolution de Montpellier UMR5554 (UM2, CNRS, IRD, EPHE), Université Montpellier 2, CC065, 34095 Montpellier, France; 2 School of Animal, Plant and Environmental Science, University of the Witwatersrand, P/Bag 3, 2050 Wits, South Africa; 3 INRA UMR CBGP, (INRA/IRD/Cirad/Montpellier SupAgro), Campus international de Baillarguet, CS 30016, F-34988 Montferrier-sur-Lez cedex, France; 4 Department of Economic Development, Tourism and Environmental Affairs, Biodiversity Research, P/Bag X20801 9300 Bloemfontein, South Africa; 5 Centre d’Ecologie Fonctionnelle et Evolutive, UMR 5175 (CNRS, UM2), Montpellier, France; 6 Department of Mammalogy, National Museum, and Centre for Environmental Management, University of the Free State, Bloemfontein, South Africa; Università degli Studi di Napoli Federico II, ITALY

## Abstract

Coexistence often involves niche differentiation either as the result of environmental divergence, or in response to competition. Disentangling the causes of such divergence requires that environmental variation across space is taken into account, which is rarely done in empirical studies. We address the role of environmental variation versus competition in coexistence between two rodent species: *Rhabdomys bechuanae* (*bechuanae*) and *Rhabdomys dilectus dilectus* (*dilectus*) comparing their habitat preference and home range (HR) size in areas with similar climates, where their distributions abut (allopatry) or overlap (sympatry). Using Outlying Mean Index analyses, we test whether habitat characteristics of the species deviate significantly from a random sample of available habitats. In allopatry, results suggest habitat selection: *dilectus* preferring grasslands with little bare soil while *bechuanae* occurring in open shrublands. In sympatry, shrubland type habitats dominate and differences are less marked, yet *dilectus* selects habitats with more cover than *bechuanae*. Interestingly, *bechuanae* shows larger HRs than *dilectus*, and both species display larger HRs in sympatry. Further, HR overlaps between species are lower than expected. We discuss our results in light of data on the phylogeography of the genus and propose that evolution in allopatry resulted in adaptation leading to different habitat preferences, even at their distribution margins, a divergence expected to facilitate coexistence. However, since sympatry occurs in sites where environmental characteristics do not allow complete species separation, competition may explain reduced inter-species overlap and character displacement in HR size. This study reveals that both environmental variation and competition may shape species coexistence.

## Introduction

The concept of character displacement is the subject of regular debate in ecology [1–4]. Ecological character displacement is defined as a process where populations respond to competition by modifying their resource-use traits through phenotypic plasticity or genetic adaptation [5]. This response to competition plays an important role in generating and maintaining biodiversity as well as shaping the mechanisms of coexistence [1,6,7], particularly between species sharing similar niches [8]. However, solid empirical evidence demonstrating the process of character displacement is rare (shown in only 9 out of 144 studies reviewed in [4]), partly due to confusion between character variation and character displacement [4,9]. Character variation due to ecological heterogeneity could occur when species adapt to distinct environments in allopatry, and may not be interpreted as character displacement when the same species are found to be divergent in sympatry [9]. Moreover, when species occur along a gradient of environmental conditions, their traits may converge in sympatry despite competition [10]. In such conditions, ecological heterogeneity across space has been argued to be a more convincing cause of character variation than competition [4].

Our study aims to test the role of adaptation to distinct environments versus competition leading to character displacement in shaping coexistence between two sister species of the African four striped mouse: *Rhabdomys bechuanae* (sensu [11], hereafter *bechuanae*) and *Rhabdomys dilectus dilectus* (sensu [12], hereafter *dilectus*). We focus here on space use, an important dimension of the niche [13] because it determines access to resources, and hence could directly influence reproductive success and survival [14]. Further, the evolution of this complex trait could be shaped both by environmental conditions [15,16] and competitive interference in areas of coexistence [17,18].

Space use, or the spatial dimension of a species niche, can be described at beta and alpha scales [19]. The beta scale considers the climate and environmental conditions over the entire range of the species defining its environmental niche. The alpha scale considers niche variation between individuals and populations (i.e. “the niche variation hypothesis”, [20]) and allows for a more detailed assessment of niche characteristics.

We studied the spatial niche of the two striped mouse species at an alpha scale by analyzing their habitat use and home range (HR) characteristics. The striped mouse shows marked differentiation across climate and vegetation along an east-to-west gradient in southern Africa [11,21]. Large scale studies, modelling the two species’ niches over South Africa and Namibia suggested environmental divergence, *dilectus* being found in the wetter areas of the north-eastern parts of South Africa where grassland vegetation dominates, while *bechuanae* occupies warmer and drier regions and penetrates into the more mesic central part of South Africa within areas where open shrubland vegetation dominates [11,21]. Such a divergence could either be the result of adaptation in distinct environmental conditions, or reflect a large range of plastic responses to the environmental gradient occupied by the two species. Here, we test the role of adaptation versus plasticity in this divergence and disentangle the role of ecological heterogeneity versus competition in shaping species coexistence in the field.

The distribution of the two species abuts in areas with similar environmental conditions, where pockets of sympatric populations exist [22]. To distinguish habitat selection from character displacement, we compared habitat preference and HR characteristics of the two species at their distribution margins where allopatric and sympatric populations are found. We made the following predictions: first, if environmental niche divergence resulted from adaptation in allopatry, as suggested by the beta scale study, populations at the margins of the two species distribution, occurring in similar environments, would select different habitats. Second, if environmental heterogeneity in areas of sympatry is sufficient to allow species segregation, we would expect little competition, if any, on the spatial dimension of the species niche. Alternatively, if environmental heterogeneity does not allow complete habitat separation, competition is expected to induce character displacement, even if sympatry is only temporary, because the trait studied here, HR size, is expected to respond rapidly to interference competition [23].

## Material and Methods

### Ethics statement

Permits to work and handle animals in the field were obtained from the Free State and North West Province reserve ethics authorities (n°01/15700, 01/11262). Animals handling was performed under permissions from the French agriculture ministry to GG (C34–265), and Wits university ethics committee for CMSD (AESC n°: 2012/13/2A).

### Study species

*Rhabdomys sp*. individuals forage alone during the day and rest at night in a nest either alone or in groups [24]. All *Rhabdomys* species are morphologically very similar, requiring genotyping for their identification. In our study, species identity was assessed by genotyping their Cytochrome Oxydase I mitochondrial gene (described in [22]).

### Study area

**Fig 1:**
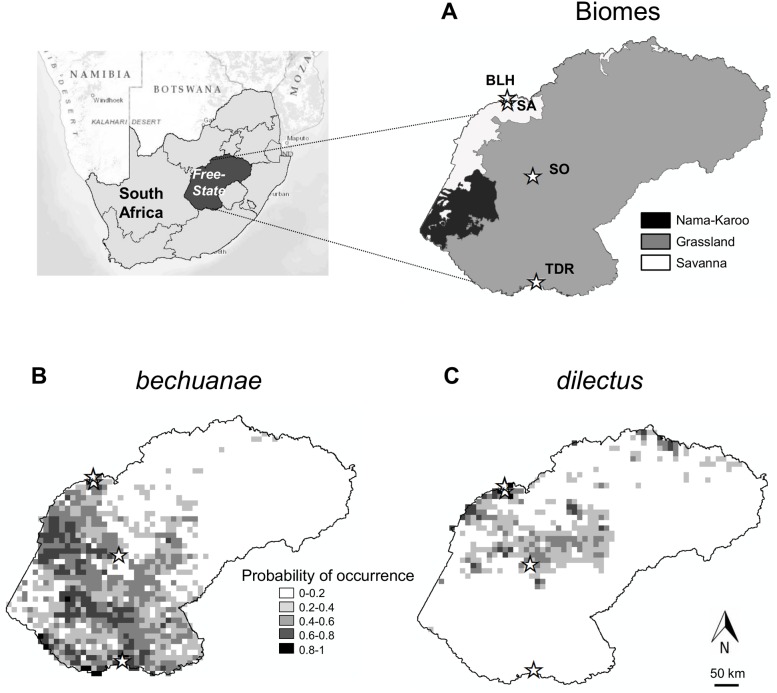
Study area and species occurrence probability. Details on biomes (A) and probabilities of occurrence of *dilectus* and *bechuanae* (B, C) (modified from Ganem *et al*. 2012). Star symbols indicate position of Bloemhof (BLH), Sandveld (SA), Soetdoring (SO) and Tussen die Riviere (TDR) Nature reserves.

Mice were studied in four nature reserves located within the savanna and grassland biomes (sensu [25]) of central South Africa: three reserves in the Free State Province and one at its boundary with the North West Province (Fig. 1). The reserves occur along a north-south axis, from Bloemhof Dam (BLH; S27° 38’ E25° 40’) and Sandveld (SA; S27° 43’ E25° 45’), to Soetdoring (SO; S28° 50’ E26° 03’) and Tussen die Riviere (TDR; S30° 28’ E26° 09’). In these reserves, *dilectus* and *bechuanae* occur in different geographic settings, either as monospecific populations (hereafter: allpatric sites) or as regular but temporary mixed species populations (hereafter: sympatric sites, S1 Fig.). In sympatric sites mice of the two species could be trapped in the same traps (although not together).

We sampled a total of 22 sites across the four reserves, among which 11 were sampled at 2 to 3 occasions (Table 1). The HRs of the mice studied never overlapped between sites during the study period (which lasted roughly two weeks per site).

**Table 1:** Characteristics of studied sites across the four nature reserves.

Reserve	Year	Season	Site	Geography	Radio-tracking	Total number of		Area (m^2^)	Percentage of area characterized with the 60x60 quadrats	Density index
						*Bechuanae*	*dilectus*			
BLH	2012	spring	BLH1	allopatry	yes	0	8	30113	75	0.03
BLH	2012	spring	BLH2	allopatry	yes	0	17	50319	31	0.04
BLH	2012	spring	BLH3	allopatry	yes	0	13	85622	20	0.01
SA	2012	spring	SA1	allopatry	yes	18	0	193027	49	0.01
SA	2011	spring	SA1	sympatry	yes	96	43	193027	49	0.06
SA	2011	spring	SA2	sympatry	yes	7	10	45531	19	0.05
SA	2012	spring	SA3	allopatry	yes	4	0	44151	31	0.01
SO	2012	spring	SO1	allopatry	yes	14	0	28150	52	0.02
SO	2012	spring	SO2	allopatry	yes	4	0	17907	53	0.01
SO	2012	spring	SO3	allopatry	yes	0	51	88514	64	0.05
SO	2012	spring	SO4	sympatry	yes	1	10	107895	42	0.01
TDR	2012	spring	TDR1	allopatry	yes	68	0	119527	48	0.07
BLH	2013	autumn	BLH3	allopatry	no	0	3	85622	20	
BLH	2012	spring	BLH4	allopatry	no	0	3	16763	52	
SA	2012	autumn	SA1	sympatry	no	25	8	193027	49	
SA	2012	autumn	SA3	allopatry	no	12	0	44151	31	
SA	2013	autumn	SA3	allopatry	no	8	0	44151	31	
SA	2013	autumn	SA4	allopatry	no	0	1	37110	48	
SA	2012	autumn	SA4	sympatry	no	4	7	37110	48	
SA	2012	spring	SA4	sympatry	no	1	2	37110	48	
SA	2012	autumn	SA5	allopatry	no	17	0	19462	45	
SA	2012	spring	SA5	allopatry	no	1	0	19462	45	
SA	2013	autumn	SA5	allopatry	no	1	0	19462	45	
SA	2012	autumn	SA6	allopatry	no	8	0	12751	71	
SA	2012	autumn	SA7	sympatry	no	7	6	124315	29	
SA	2012	spring	SA7	sympatry	no	5	1	124315	29	
SA	2012	autumn	SA8	allopatry	no	4	0	7659	70	
SA	2012	autumn	SA9	allopatry	no	18	0	43011	34	
SA	2013	autumn	SA9	allopatry	no	5	0	43011	34	
SO	2012	autumn	SO1	allopatry	no	4	0	28150	52	
SO	2012	autumn	SO3	allopatry	no	0	52	88514	64	
SO	2012	autumn	SO4	allopatry	no	0	11	107895	42	
SO	2012	autumn	SO5	allopatry	no	7	0	18942	33	
SO	2012	autumn	SO6	allopatry	no	0	1	6537	47	
SO	2012	autumn	SO7	sympatry	no	1	1	9503	64	
TDR	2013	autumn	TDR1	allopatry	no	4	0	119527	48	
TDR	2012	spring	TDR2	allopatry	no	3	0	17701	57	

BLH: Bloemof, SA: Sandveld, SO: Soetdoring and TDR: Tussen Die Riviere nature reserves.

Although we did not monitor predation and competition with other species, we observed the presence of snakes, birds of prey and carnivore small mammals in all sites. Other rodent species were present in the trapping sites: the most frequent ones were *Gerbiliscus sp*. and *Nannomys minutoides*.

### Trapping procedure

Trapping took place during the austral spring: October-November 2011 (SA), October-November 2012 (SA, SO, TDR, BLH), and autumn: April-May 2012 (SA, SO) and April-May 2013 (SA, TDR, BLH). Our trapping strategy aimed to sample most vegetation types available within the area. Small mammal live traps (Sherman and PVC traps of equivalent size) were baited with a mixture of oats, salt and peanut butter, and were provided with a piece of cotton wool as bait and to reduce thermal stress. The number of trap lines varied with the site size, and distance between traps was roughly 10m (10 to 30 traps/line). Traps were checked 2–3 times a day regularly between 7am and 7pm (mean±SD: 79.5±50.89 traps per day per site). Upon capture, each mouse was sexed, weighed, measured (body length) and individually marked with two ear tags (7mm, 0.17g; National Band and Tag Co., Newport, KY-USA). We also collected a piece of tail (≈1cm) for species identification. Overall, we trapped and genotyped 599 mice. Following [26], we estimated relative density within each site by computing the total number of striped mice captured during the first five trapping days divided by the total length of trap lines accounting for a 60 m buffer around the trap lines (roughly the average diameter of a mouse HR, Table 1).

### Habitat characterization

Earlier studies addressing beta scale niche analysis [21,22] suggested that the two species could have different requirements in terms of vegetation cover and structure (i.e. grass versus woody vegetation). Here we aimed to test this hypothesis at an alpha scale, and characterized the vegetation structure of mouse habitats by measuring the percentage of trees, bushes, grass and bare soil over 60x60m quadrats, centred on a trap line (the first and last traps were at the centre of, respectively, the first and last quadrats on a given trap line). Furthermore, vegetation cover was determined within 1x1m metal square thrown to the right and left of a trap line at every second trap. Within these 1x1m quadrats, we evaluated the percentage of grass versus woody plants (small shrubs), the percentage of bare soil and an estimate of mouse visibility (an index ranging from 1, i.e. completely visible, to 5, i.e. completely hidden, a value determined by averaging the visibility of a dummy mouse that we placed in four different locations inside the metal square). Altogether we used 136 60x60m quadrats (5.9±6.31 per site) and 229 1x1m quadrats (10.4±15.37 per site).

### Radiotracking

A total of 101 adult mice (body mass ≥ 23 grams), were equipped with VHF collars (MD 2C Holohil, Carp, Ontario, Canada) in October-November 2011 and 2012 in 11 distinct sites on the four reserves. Radiotracking was performed on foot with a wide-range receiver (AOR 8000) and a hand-held Telonics R4–14K antenna. Localization of a collared mouse followed the standard triangulation technique and its precise location was confirmed with the receiver cable used without the antenna. The receiver volume was set to 0 during the triangulation to reduce mice disturbance. The GPS coordinates of radio-collared individuals were recorded five times during the day (at about 7, 9, 11am and 2 and 4pm) and once at sunset (roughly 7pm).

### Home range size and overlap estimations

HRs were defined as the areas encompassed within the 0.95 cumulative isopleth of the Utilization Distributions (UDs), estimated using the fixed kernel method with the reference smoothing parameter [27]. Our sampling regime was chosen after a calibration session where we followed 30 individuals for more than seven days (from 40 to 69 relocations). The estimated HR size of our controls stabilized after 27 relocations and a paired comparison of the HR size at 27 and 41 regular relocations did not show a significant difference (Wilcoxon test, V = 306, p = 0.14). Following [28], we chose a strategy maximizing the number of mice radiotracked with a sampling regime standardized to a minimum of 27 independent relocations, collected over five consecutive days.

We compared HR overlaps in a sympatric site between pairs of mice of the same species versus different species. We computed the overlap between each pair of HRs using their UD-based volume of intersection [29]. Because UDs are truncated at the 0.95 cumulative isopleth (excluding the poorly estimated UD tails), overlap values were normalized to 1 by dividing them by 0.95 (see [30] for details).

### Statistical analyses

Statistical analysis was conducted with R-v2.15 [31]. Normality and heteroscedasticity of distributions were checked with a Shapiro test and visualized with the plot of the model’s residuals. When these conditions were not met even after data transformation, non-parametric tests were used. Significance level was set to 0.05, and adjusted for multiple comparisons with the sequential Bonferroni procedure when necessary. UD, overlap computations, and permutation tests (see below) were performed using home-made programs in Pascal.

#### Mice-habitat relationship assessed with trapping data

A total of 599 trapped mice were used in these analyses. A mouse was considered as potentially using a 60x60m quadrat when it was trapped within it, and a 1x1m quadrat when it was trapped less than 10m from it. Each quadrat was then assigned to one or the other species, to both species, or to none. A total of 89 60x60m quadrats and 227 1x1m quadrats were assigned to one or the two species.

We performed an Outlying Mean Index multivariate analysis to characterize the environmental niche of each species (i.e. OMI) [32]. Briefly, the OMI procedure generates ordination axes corresponding to the combination of environmental variables (here vegetation structure and cover) that are most relevant for the species under study, and provides a measure of the habitat conditions occupied by the species. Our two habitat parameters (vegetation structure and cover) were obtained at different sampling scales, hence, we carried out an OMI analysis at each scale (i.e. 60x60m and 1x1m). Each analysis produced a habitat niche position value (i.e. the mean habitat characteristics of species occurrence) and breadth (i.e. variance) for each of the four categories studied here, i.e. the two species in allopatric versus sympatric sites. We assessed marginality (i.e. deviation from a random sample of available conditions) of niche position and breadth of a given category on an OMI axis through comparisons with distributions obtained performing 1000 random permutations followed by bootstrap two-tailed tests.

The niche positions of the four population categories (two species in allopatry versus in sympatry) on the first OMI axis (OMI1) were compared with linear mixed ANOVAs (package nlme), with the category as a fixed effect and site as a random effect, followed by Tukey post-hoc tests when relevant (package mulcomp, glht function). The same procedure was applied on a subsample of the data comprised of only sympatric sites, to test whether excluding allopatric habitats from the analyses would detect species divergence in sympatry.

#### Mice-habitat relationships assessed with home range data

Habitat at the HR scale was characterized with the 60x60m quadrats that covered an area corresponding to at least 70% of the HR. Such a coverage was reached for 80 of the estimated HRs (S1 Table). The four vegetation structure variables measured within a quadrat were weighted by the relative proportions of the HR UD covered. These data were then analyzed following the same procedure as described above.

#### Determinants of home range size variation

For the purpose of this analysis, we reduced the four variables describing vegetation structure into one corresponding to the first axis of a Principal Component Analysis (PCA). This axis represented 76% of total variance. We tested the influence of body size, sex, population density, habitat (i.e. PCA1), geography (i.e. allopatry vs sympatry) and species on log-transformed HR size. Our data showed spatial autocorrelation (Moran test, p<0.001, library “spdep”), hence we applied the spatial simultaneous autoregressive error model estimation (sarlm model) in subsequent ANCOVA analyses. Our initial ANCOVA model comprised all factors as main effects and second and third order interactions with “species”, except for density which was included as a co-variable in the model. Preliminary analyses showed that density did not vary between species (KW, DF = 2, χ^2^ = 1.72, p = 0.42) or with the habitat parameter of the model (Spearman, ρ = 0.06, p = 0.58), and that body size did not differ between our sample of males and females (Anova, DF = 1,97, F = 0.61 p = 0.44). The most parsimonious model was obtained after sequential elimination of factors with non-significant effects (following [33]), and post-hoc checking that its AICc was significantly smaller to that of the initial model.

#### HR overlaps in a sympatric site

Because we did not know the species identity of mice during our field study, our selection of radiotracked mice could not be balanced. The between-species HR overlap analysis could eventually be performed for only one sympatric site (SA1), in which 6 *dilectus* (3 females and 3 males) and 14 *bechuanae* (9 females and 5 males) were radiotracked. Other sympatric sites contained too few radiotracked individuals of one species or the other to enable us to perform statistical tests. We computed three observed values: the mean overlap between any two *dilectus* HRs, the mean overlap between any two *bechuanae* HRs and the mean overlap between HRs of any two mice belonging to different species. To test the null hypothesis: “random overlap between species”, we determined all the possible partitions of 20 mice in a group of 6 (G6) and a group of 14 (G14). Because the degree of HR overlap between two mice could also depend on sex, we kept only the 12320 partitions (out of 38760) showing the observed sex ratios (i.e. 3 females and 3 males in G6 and 9 females and 5 males in G14). For each partition, we computed the mean overlap between two mice belonging to the same group (G6xG6 and G14xG14) or to different groups (G6xG14). Mean values are based on 15 (G6xG6), 91 (G14xG14) or 84 (G6xG14) observations. In this way, by considering the whole set of partitions, we built up three theoretical distributions expected under the null hypothesis, to which we compared the three observed values mentioned above. As in any permutation test, the probability to reject the null hypothesis, i.e. obtaining a value equal to or more extreme than the observed value in each of the three tests, was computed as *P = 2*(*n*_*e*_+1)/*n* (bilateral test), where *n*_*e*_ is the number of these most extreme values and *n* is the total number of values (*n* = 12320).

## Results

### Variation of habitat preference within and between species

**Fig 2:**
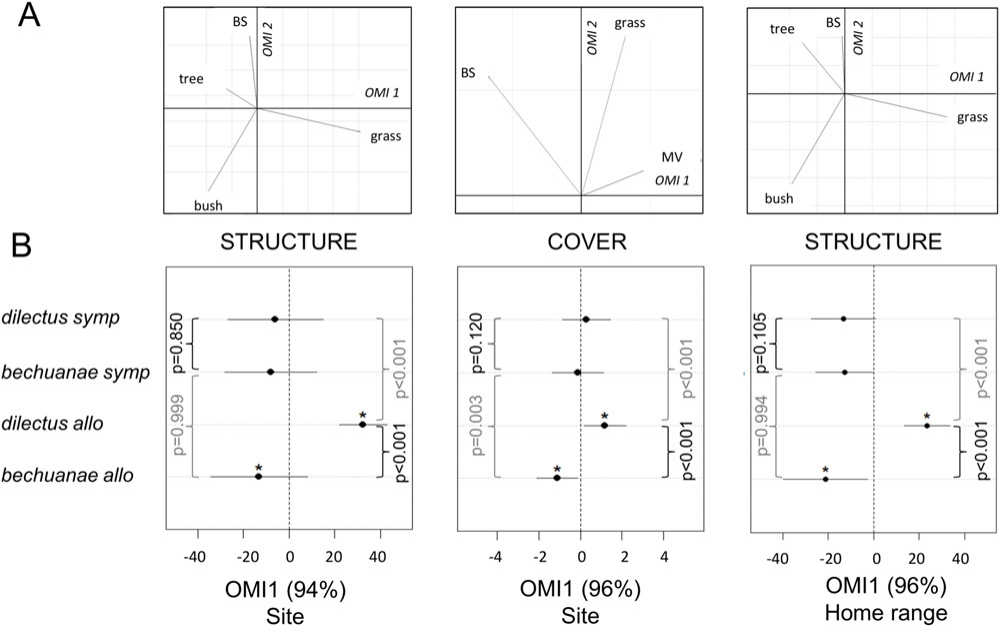
Habitat niche divergence in sympatry and allopatry. Habitat divergence between allopatric (allo) and sympatric (symp) populations of the two species as assessed with Outlying Mean Index (OMI) analyses. The upper row (A) shows the relative contribution of the different habitat variables: vegetation structure (tree, grass, bush and bare soil (BS)) and cover (bare soil (BS), grass and mouse visibility (MV)) to the first two OMI axes. The lower row (B) indicates the position (dot) and breadth (line) of each species niche along the first OMI axis (* when significantly different from random expectation). The p-values of Tukey tests are given for every pair comparison (black: inter-specific, grey: intra-specific).

We compared the habitat characteristics of allopatric and sympatric mice of the two species at the population (site) and individual (HR) levels with three distinct OMI analyses. The first axes (i.e OMI1) always captured a significant proportion of the habitat variation (>94%, Fig. 2), and results were consistent across analyses. These three axes had positive values for presence of grass and mouse visibility, and negative values for the presence of woody type vegetation and bare soil (Fig. 2). The habitat niche positions of the two species in allopatric sites were significantly different from a random sample of available habitats (p<0.05), and from each other (respectively, at the site level, vegetation structure and cover, and at the HR level vegetation structure: z = -4.41, z = -7.69 and z = 5.28, p<0.001). The niche position of allopatric *bechuanae* showed significant negative values (i.e. habitat characterized by more woody vegetation and presence of bare soil than that in a random sample of available habitats), while those of *dilectus* were significantly positive (i.e. habitats characterized by the presence of more grass and cover than in a random sample). Unlike allopatric sites, the niche position of individuals of the two species in sympatry did not differ from random expectations (p>0.05). However, their positions differed from that of their allopatric counterparts. Indeed, in sympatry, *bechuanae* occurred in a habitat with higher mouse visibility and cover values (vegetation cover, z = 3.39, p = 0.003), but with similar values of vegetation structure (at the site and HR levels, Fig. 2) compared to its habitat in allopatry. In contrast, habitat characteristics of *dilectus* in allopatry and sympatry differed in the three analyses, as its habitat in sympatry was characterized by lower values of mouse visibility, cover and presence of grass than in allopatry (respectively, at the site level the vegetation structure and cover, at the HR level the vegetation structure: z = -3.88 z = -3.84 z = -4.29 p<0.001, Fig. 2).

**Fig 3:**
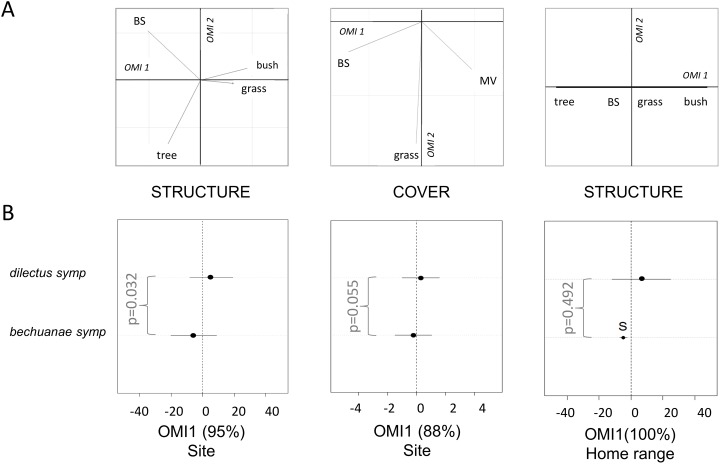
Details on habitat niche divergence in sympatry. Habitat divergence of the two species in sympatric sites assessed with Outlying Mean Index (OMI) analyses. See legend in Fig. 2. The p-values follow Wilcoxon tests.

Considering only sympatric sites, the first OMI axes captured most of the data variation (>88%), and described a habitat gradient ranging from high values of bare soil and presence of trees (negative values of OMI1) to high values of grass, mouse visibility and presence of bushes (positive values of OMI1, Fig. 3). Despite the reduced power due to a smaller sample size (particularly for the HR level analysis), the results indicate that *bechuanae* occurs in micro-habitats characterized by more bare soil, woody vegetation and less mouse visibility than that of *dilectus*, although these differences (respectively at the site level, the vegetation structure and cover: p = 0.03 p = 0.05) were not strong (significance level adjusted for multiple testing α’ = 0.025; Fig. 3).

### Variation of HR size within and between species

**Fig 4:**
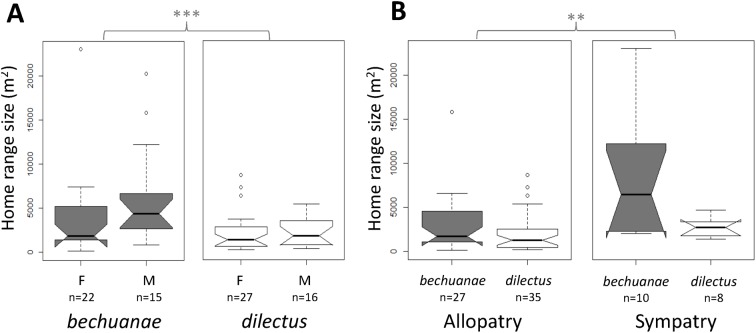
Home range size estimates. Home range size estimates (isopleth 0.95) across species (grey: *bechuanae*, white: *dilectus*), A: sex (F females and M males) and B: “geography” (** p<0.01, *** p<0.001 refers to Ancova results in S2 Table). Box-plots show the median (thick line), first and third quartiles. Non-overlapping notches are roughly equivalent to 95% confidence intervals.

None of the first and second order interactions with species were significant predictors of HR size variation, indicating that the patterns described below were consistent across species. Males had larger HRs than females (z = 2.37, p = 0.02, Fig. 4, S2 Table) and population density and habitat did not significantly affect HR size (respectively, z = -0.89, p = 0.37 and z = 1.66, p = 0.10, S2 Table). Further, *bechuanae* had larger HRs than *dilectus* (z = 3.40, p<0.001) both in allopatry and sympatry, although the HRs of both species were smaller in allopatry than in sympatry (z = 2.82, p<0.01, Fig. 4, S2 Table).

### Patterns of overlap in a sympatric site

Overlaps of HRs between the two species were significantly lower than random expectations (observed value: 0.011±0.048; permutation test p<0.01), while they were higher than expected within *bechuanae* (observed value: 0.057±1.265; p<0.01), and not different from random within *dilectus* (observed value: 0.023±0.052; p>0.5, S2 Fig.).

## Discussion

Our study is among very few that attempted to disentangle the complex interaction between environment and competition in shaping character variation [23]. We focused on the spatial niche (i.e. habitat selection and HR size) of two sister species of striped mice whose distributions are mostly allopatric and characterized by distinct environmental conditions [11,12]. We assessed the influence of environmental variation and competition on habitat selection and HR size variation in an area where the distributions of the two species abut and where allopatric and sympatric populations can be compared under similar climatic conditions [22].

Earlier large scale investigations indicated that the environmental niche of the two studied species diverged: the habitats of *bechuanae* are dominated by warmer climates and drier open shrubland vegetation, while those of *dilectus* are characterized by wetter climates and grassland type vegetation, providing more cover [11,21,22]. Such beta scale studies are particularly relevant to address species niche characteristics over their entire range; however, they could suffer from confounding effects due to spatial autocorrelation of large scale environmental variables and are not expected to reveal micro-environmental heterogeneity [34], but see [35]. Nevertheless, using an alpha approach, the present study confirms habitat niche divergence at a fine scale and highlights a role for both environmental variation and competition in shaping the spatial niche of *bechuanae* and *dilectus* in sympatry.

### Niche divergence

#### Habitat

Sister species are expected to have similar niches if they retain ancestral characteristics (niche conservatism, [36]), or when they evolve under similar conditions [37]. In all cases, contact or secondary sympatry between species sharing similar niches are expected to trigger character displacement [8,35]. Earlier studies of the striped mouse environmental niche indicated that evolution in allopatry took place under different environmental conditions [11,21], and that divergent adaptation may facilitate coexistence. The present study confirms divergence in habitat selection by the two species at the margins of their distributions but also indicates that the available habitat in sympatry is more similar to that of *bechuanae* in allopatry (i.e. less cover and more woody vegetation) than that of *dilectus*. Nevertheless, the latter still selects micro-habitats with more cover and less woody vegetation than *bechuanae*, confirming micro-habitat partitioning in sympatry as suggested by preliminary observations [22]. Such differences in habitat preference, consistent over the entire species range including its margins and sympatric zones, together with the largely allopatric distribution of the species, support evolution under different selective pressures in allopatry (i.e. adaptation), and a more recent secondary contact where these preferences are still expressed. Partition of the habitat niche could thus result in lower interspecific competition pressures facilitating co-existence [38,39]. Nevertheless our study indicates that habitat divergence in sympatry is tenuous compared to that in allopatry. Further, the habitat available in sympatry differed significantly from that in allopatry for *dilectus*, suggesting that the latter invaded the range of *bechuanae* and that coexistence occurs in areas to which *dilectus* might be less adapted.

#### Home Range

Lesser habitat partition in sympatry is expected to induce competition which we assessed comparing HR size variation, a trait known to be influenced by habitat features [16] and interspecific interference [40]. We found differences in HR size between the species: *dilectus* having smaller HRs than *bechuanae*. These differences exist despite sexual dimorphism in HR size (i.e larger in males than females), in both species, that could relate to behavioural [41,42] and physiological [16,43] sex differences.

Our study did not address the precise mechanisms of HR divergence; however, based on inference from the literature, we may expect that, like other species, striped mice adjust their HR size to available cover or shelter (e.g. [16] on the wood mouse) or to visibility of potential predators (e.g. [44] on roe deer). Such patterns are consistent with our observations that, the species showing preference for habitats with cover, *dilectus*, also has smaller HRs compared to *bechuanae* which selects habitats with more wood and less cover. Larger HRs may also provide access to patchily located shelters from predators [45], which may be the case for *bechuanae* which selects open shrubland type habitats. Differences in HR size may also indicate differences in food distribution, since smaller HRs were proposed to reflect more concentrated food distribution in other studies [44,46–49]. Surprisingly, we did not detect a significant influence of vegetation structure on HR size variation. Possible explanations might be that, either this variable only has an indirect effect on HR size (see above), or to lower resolution due to small sample size.

### Character displacement

Differences in habitat preferences may facilitate coexistence between *bechuanae* and *dilectus*. However, as indicated above, sympatry occurs in habitats that are less favorable for *dilectus* and our study suggests that species segregation in sympatry may not be complete. Patterns of HR size variation in allopatry versus sympatry also suggest that competition may occur. Indeed, both species had a significant increase in their HR size in sympatry as compared to allopatry. Although we cannot exclude that such variation could be consistent with habitat variation as far as *dilectus* is concerned, this argument may not hold for *bechuanae* whose preferred habitat in allopatry and sympatry is similar.

A larger HR size in sympatry may reveal inter-species intolerance and competition [17,23,40]. In our study, larger HRs in sympatry compared to allopatry, at least in *bechuanae*, could be a response to competition and a strategy aimed at limiting costly interactions with the other species through character displacement [40]. Alternatively, it could be a strategy to occupy most of the available resources (e.g. nest sites). Patterns of HR overlap in our study suggest that our first hypothesis may be true, as *bechuanae* showed more HR overlaps than expected with members of the same species, while between species overlaps were lower than expected under random predictions.

Micro-habitat selection and space partition are expected to be adaptive responses to reduce competition [17,18,50]. Here, despite different habitat preferences, habitat segregation is tenuous in sympatry, resulting in *bechuanae* enlarging its HR possibly to avoid *dilectus*, making detours, or to control a larger number of shelters to outcompete *dilectus*.

## Conclusion

It was argued that ecological complexity was not considered often enough in assessments of mechanisms of coexistence [9], and that evidence for character displacement resulting from species interference is rare [4]. Our study provides valuable field data in an interesting study model allowing to compare the spatial niche characteristics of two species in a relatively homogeneous sympatric and allopatric environment (a common garden setting). Furthermore, the alpha scale investigation, together with an earlier beta scale one [11,21,22], provides a comprehensive picture of how environmental heterogeneity and interference competition could shape the spatial niche of two sister species and influence patterns of coexistence. Future studies should include mechanistic experimental approach to address competition between the two species and determine the proximal mechanisms (e.g. the impact of competition on the species fitness) shaping the species range limits and patterns of co-existence.

## Supporting Information

S1 Fig Experimental design. A: An example of distribution of allopatric and sympatric sites (SO1-SO6) within Soetdoring Nature Reserve. B: Distribution of the quadrats used for habitat assessment (vegetation structure and cover) around the trap lines.
(TIF)

S2 Fig Three theoretical distributions of the mean volumes of interaction within HR overlaps. From left to right: distributions of intra-species overlap values within *dilectus* (A) and *bechuanae* (B) and between the species (C). The red lines indicate position of observed mean values.
(TIF)

S1 Table HR estimates sample size. Number of HR estimates per species and sex in allopatric and sympatric sites, and size of the subsample for which the habitat was characterized.
(XLSX)

S2 Table Factors influencing HR size variation. Results of the initial and minimal ANCOVA models. The models (sarlm residual, see text), testing factors that may influence HR size variation: habitat (PCA 1), sex, geography (allopatry vs sympatry), body size and population density. Bold p-values indicate significant effects.
(XLSX)
