# Supplementary figures and images for: Space Use Variation in Co-Occurring Sister Species: Response to Environmental Variation or Competition?
Source: PLoS One. 2015 Feb 18;10(2):e0117750. doi: 10.1371/journal.pone.0117750 (PMC4333358; doi:10.1371/journal.pone.0117750)

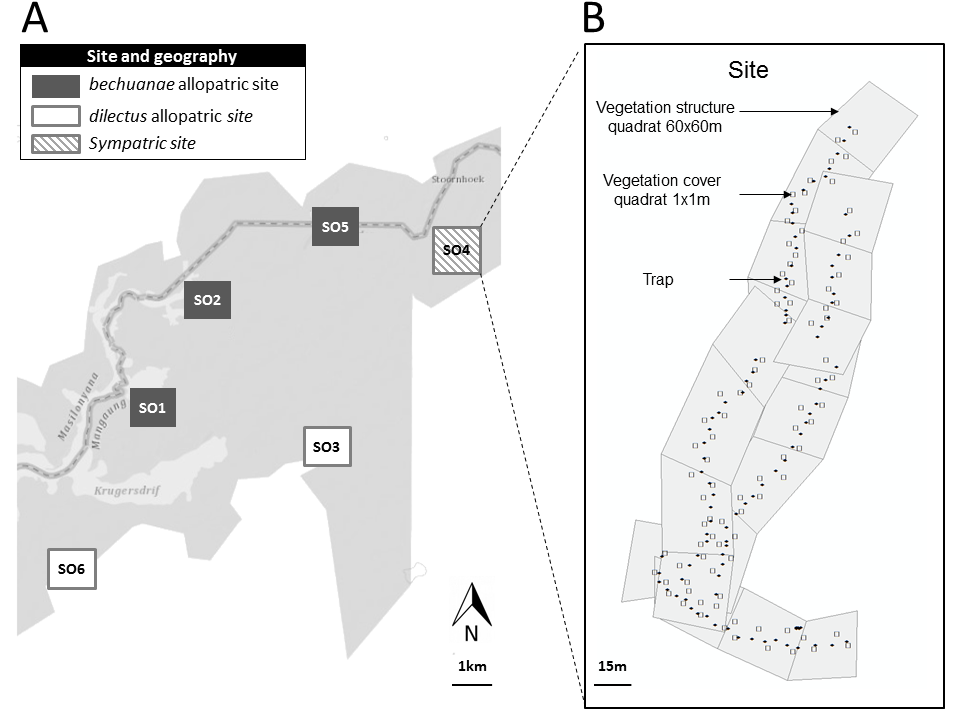

Supplement: S1 Fig — A: An example of distribution of allopatric and sympatric sites (SO1-SO6) within Soetdoring Nature Reserve. B: Distribution of the quadrats used for habitat assessment (vegetation structure and cover) around the trap lines. (TIF) [file pone.0117750.s001.tif]

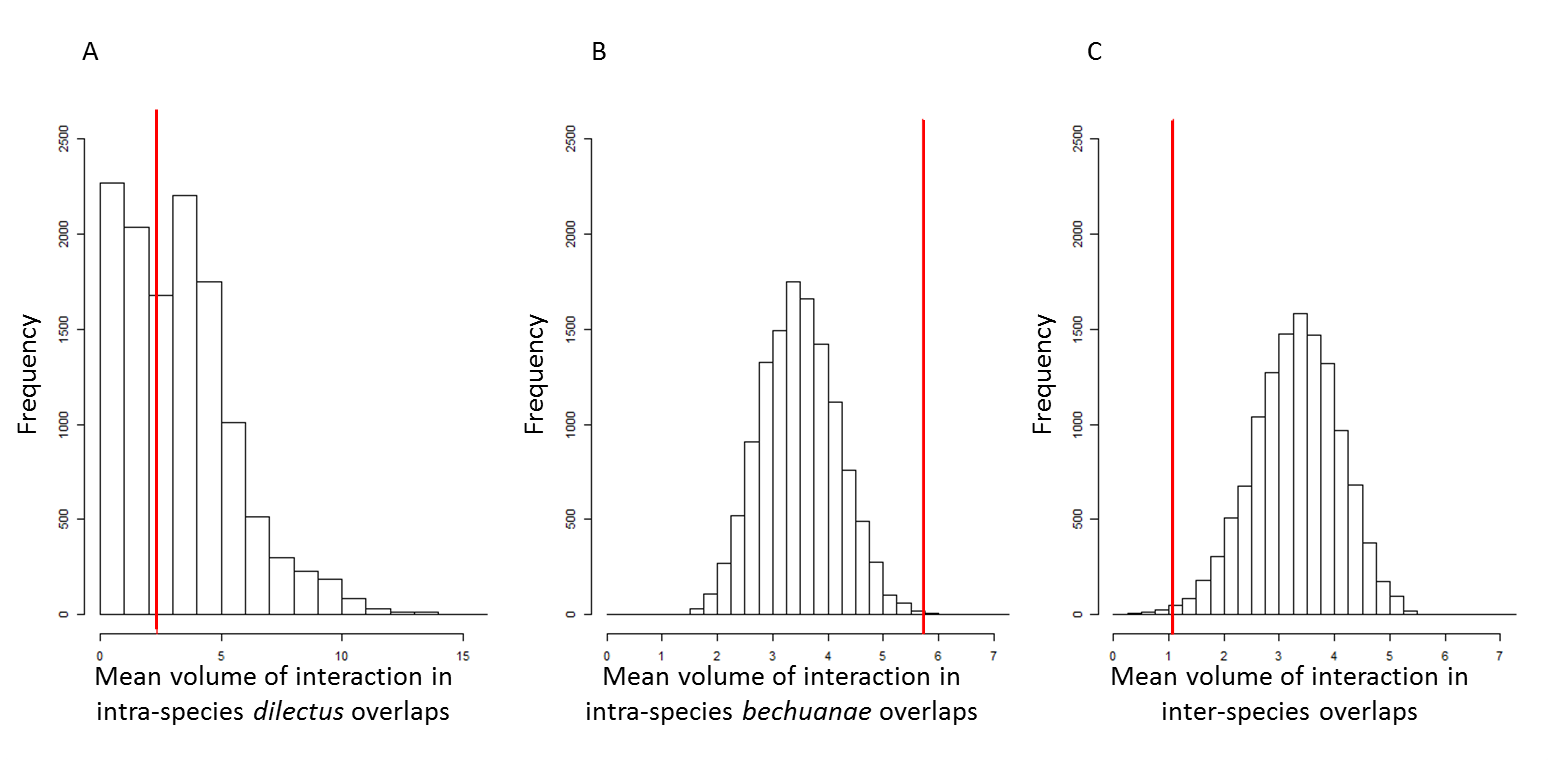

Supplement: S2 Fig — From left to right: distributions of intra-species overlap values within dilectus (A) and bechuanae (B) and between the species (C). The red lines indicate position of observed mean values. (TIF) [file pone.0117750.s002.tif]
